# Changes in Body Composition Are Associated with Metabolic Changes and the Risk of Metabolic Syndrome

**DOI:** 10.3390/jcm10040745

**Published:** 2021-02-13

**Authors:** Yun Hwan Oh, Seulggie Choi, Gyeongsil Lee, Joung Sik Son, Kyae Hyung Kim, Sang Min Park

**Affiliations:** 1 Department of Family Medicine, Jeju National University Hospital, Jeju 63241, Korea; swimayo@gmail.com; 2 Department of Family Medicine, School of Medicine, Jeju National University, Jeju 63243, Korea; 3 Department of Biomedical Sciences, Graduate School, Seoul National University, Seoul 03080, Korea; seulggie@gmail.com; 4 Department of Family Medicine, Seoul National University Hospital, Seoul 03080, Korea; gespino1.gs@gmail.com (G.L.); medical114@naver.com (J.S.S.); kyaehyungkim.snuh@gmail.com (K.H.K.); 5 Comprehensive Care Clinic, Seoul National University Hospital, Seoul 03080, Korea

**Keywords:** metabolic syndrome, body composition, lean body mass, appendicular skeletal mass

## Abstract

In a cohort of 190,599 participants from The National Health Insurance Service-National Health Screening (NHIS-HEALS) study, we investigated the association of changes in the predicted body composition and metabolic profiles with the risk of metabolic syndrome (MetS) in the general population, which was hitherto incompletely elucidated. At baseline and follow-up examinations, the body composition, including lean body mass (LBM), body fat mass (BFM), and appendicular skeletal mass (ASM), were estimated using a prediction equation, and the risk of MetS was analyzed according to relative body composition changes. An increase in relative LBM and ASM decreased the risk of MetS in men and women (adjusted odds ratio (aOR), 0.78 and 0.80; 95% confidence interval (CI), 0.77–0.79 and 0.79–0.81, respectively; all *p* < 0.001). As relative LBM and ASM increased, the risk of MetS was more significantly reduced in the group with higher baseline BMI and body fat mass index (BFMI)(all *p*-trend < 0.001). In men, when the relative LBM increased (aOR, 0.68; 95% CI, 0.63–0.73), the risk of MetS was low despite increased BMI. Thus, our findings suggested that an increase in the relative LBM and ASM reduced the risk of MetS, whereas an increase in the relative BFMI increased the risk of MetS; this result was consistent in men despite an increase in BMI.

## 1. Introduction

Metabolic syndrome (MetS) is a pathological condition characterized by abdominal obesity, impaired fasting glucose, dyslipidemia, and hypertension. It is estimated that approximately one-third of the US population, 15.5% of China’s population, and approximately one-quarter of the world population have MetS [1]. In addition to the widespread global prevalence of MetS, the associated increase in the risk of developing several other diseases, including atherosclerotic cardiovascular disease, type 2 diabetes [2,3], and cancer [4] in patients with MetS, deserves close attention.

Body composition is related to various physiological and pathological states. Excessive body fat, which is characteristic of obesity, is a major health risk. Several organizations, such as the World Health Organization [5], the National Cholesterol Education Program-Adult Treatment Panel III (NCEP-ATP III) [6], the International Diabetes Federation [7], and the American Association of Clinical Endocrinologists [8], have proposed abdominal adiposity as the main parameter to determine MetS. Furthermore, it is well established that adiposity plays a major role in the development of associated diseases [9,10].

Body mass index (BMI), which is the preferred measure to assess general obesity, actually does not fully represent the adiposity of the body. The BMI does not distinguish the different body components of fat mass and muscle mass. Recently, the general clinical perception that the lack of muscle mass and excessive body fat are associated with metabolic diseases has increased [11]. Moreover, the lack of muscle mass is recognized as a serious health risk. Sarcopenia is characterized by a progressive and generalized loss of skeletal muscle mass and strength [12]. Sarcopenia is not only a change in body composition in the course of the natural aging process but also the pathologic status associated with a broad spectrum of metabolic disorders, including MetS [13], diabetes mellitus [14], and cardiovascular disease [15,16].

It is well-known that weight reduction can reduce the risk of MetS and improve metabolic profiles [17]. However, body weight is composed of both body fat mass and muscle mass. Based on the current state of knowledge, interventions are mainly focused on a reduction of body fat for managing obesity. Despite the different effects of body fat mass and muscle mass on metabolic health, no study has investigated the impact of changes in muscle mass and body fat mass at the nationwide population scale. A reason for this gap may be the limitation in adapting accurate modalities, such as dual X-ray absorptiometry or bioimpedance analysis, for measuring body composition in large-scale epidemiologic studies. Therefore, we adopted prediction equations for calculating the participant body compositions. In this study, using the prediction equation, we aimed to investigate the association between the changes in the predicted body composition and changes in the metabolic profiles and the risk of MetS in a large Korean population.

## 2. Methods

### 2.1. Participants

The Korean National Health Insurance Service (NHIS) database provides health insurance to >95% of Korean citizens [18] and biannual health screening examinations for all Korean citizens aged ≥40 years. The health examination consists of self-reported questionnaires, basic physical examination results, and blood investigations for biochemical markers. Self-reported questionnaires comprise participant health behaviors, medical history, and family history. The basic physical examination results contain information on height, weight, waist circumference (WC), and blood pressure (BP). Blood investigations included various biochemical markers, such as fasting serum glucose (FSG), triglyceride (TG), high-density lipoprotein cholesterol (HDL-C), and serum creatinine levels. The National Health Insurance Service-National Health Screening Cohort (NHIS-HEALS) followed up men and women aged ≥40 years who underwent health examinations between 2002 and 2015. Several studies have used the NHIS-HEALS database for epidemiological studies, and the validity of the database has been described elsewhere [19].

**Figure 1:**
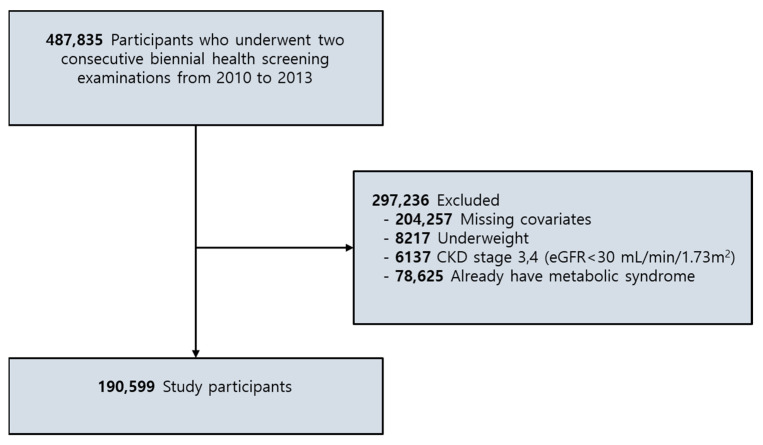
Flow diagram showing the selection of study participants. CKD, chronic kidney disease. eGFR estimated glomerular filtration rate.

Of the 487,835 participants who underwent health examinations during the first (2010–2011) and the second (2012–2013) periods, 204,257 participants were excluded because of missing information on anthropometric profiles, metabolic profiles (including WC, BP, FSG, TG, and HDL-C), physical activity, smoking status, alcohol habit, and serum creatinine levels. In total, 8217 underweight participants (BMI < 18.5 kg/m^2^) were excluded from the analysis, as were 6137 participants with an estimated glomerular filtration rate of <30 mL/min/1.73 m^2^, consistent with chronic kidney disease stages IV and V. An additional 78,625 participants who already had MetS at baseline were excluded. Finally, 190,599 participants, including 101,536 men and 89,063 women, were enrolled. Figure 1 shows the participant-selection flow diagram of this study.

### 2.2. Key Variables

#### 2.2.1. Body Composition

A previous study [20], using a representative sample of the Korean population from the Korean National Health and Nutrition Examination Survey (KNHANES), included multivariable linear regressions to derive the prediction equations for lean body mass index (LBMI), body fat mass index (BFMI), and appendicular skeletal mass index (ASMI). The following variables were used to derive the prediction equations: LBM, ASM, and BFM from whole-body dual-energy X-ray absorptiometry as dependent variables; and age, anthropometric measurements (height, body weight, and WC), blood test (serum creatinine levels), health-related behaviors (physical activity level, smoking status, and alcohol intake) as independent variables that can be related to factors that affect the body composition. Based on their results, the authors suggested prediction equations for the assessment of body composition, including LBMI, BFMI, and ASMI. We adapted these prediction equations to estimate the participants’ body composition in this study.

The prediction equations were validated using the Bland–Altman plot and the intraclass correlation coefficient (ICC). The ICCs were all >0.9. Each of the prediction equations showed a high predictive power for each body composition [20] (LBMI, ASMI, and BFMI) and a low bias in the Bland–Altman plot, with good agreement between the actual measured and predicted values estimated by the suggested prediction equation when applied in large-scale population studies. In this study, each participant’s LBMI (kg/m^2^), BFMI (kg/m^2^), and ASMI (kg/m^2^) were calculated using the prediction equations presented in the Supplementary Data.

#### 2.2.2. Changes in Body Composition

Participant LBMI, BFMI, and ASMI were calculated for each of the two health examination periods and converted into the percentage lean body mass (LBMI/BMI × 100), percentage body fat mass (BFMI/BMI × 100), percentage appendicular skeletal muscle mass (ASMI/BMI × 100), relative lean body mass (relative LBM), relative body fat mass (relative BFM), and relative appendicular skeletal muscle mass (relative ASM). The relative LBM, BFM, and ASM were adopted in this study because these parameters could be adjusted for the body frame size. Additionally, the changes in relative body composition were the main focus of this study. The change in the relative LBM, termed ΔRelative LBM, was calculated from the difference between the first and second relative LBM, and the ΔRelative BFM and ΔRelative ASM were also calculated similarly.

#### 2.2.3. Metabolic Syndrome

MetS was determined based on the NCEP-ATP III criteria that were modified for the Asian population [21]. The participants were diagnosed with MetS if they met three of the following five conditions: (1) a WC ≥ 90 cm (for men) or ≥85 cm (for women) [22], (2) TG levels ≥ 150 mg/dL or treatment with lipid-lowering drugs, (3) HDL-C levels < 40 mg/dL (men) or < 50 mg/dL (women), (4) BP ≥ 130/85 mmHg or treatment with antihypertensive drugs, and (5) FSG levels ≥ 100 mg/dL or antidiabetes pharmacotherapy. The presence of MetS was determined for each participant during the initial and follow-up health examinations.

#### 2.2.4. Other Variables

Smoking status, alcohol consumption, and physical activity were assessed by using a self-reported questionnaire. According to the previous study that described the characteristics of the NHIS-HEALS cohort database, the questionnaires for evaluating smoking status and alcohol use status were composed as follows [23]: (1) smoking status: cigarette smoking status (current smoker, ex-smoker, never smoker), the current daily smoking dose for a current smoker, past daily smoking dose for ex-smoker, smoking duration (2) alcohol use status: days of drinking per week, amount of drinks per occasion. As regards smoking status, the participants were categorized into three groups: never smokers, ex-smokers, and current smokers. Further, the status of alcohol consumption was categorized as none, moderate, and heavy. Heavy alcohol intake was defined as ≥14 drinks and ≥7 drinks per week for men and women, respectively. This heavy alcohol intake definition is the same as the Centers for Disease Control and Prevention (CDC) definition [24]. The drinks were calculated by multiplying the average drinking frequency per week by the number of drinks per occasion. The physical activity was assessed using the Korean version of the International Physical Activity Questionnaire (IPAQ) [25]. The total magnitude of physical activity was calculated based on the metabolic equivalent task (MET)-minutes/week (walking, 3.3 METs; moderate physical activity, 4.0 METs; and vigorous physical activity, 8.0 METs). Each participant’s physical activity was categorized based on the total physical activity METs and the frequency of each type of physical activity according to the IPAQ scoring guide [26]. The Charlson comorbidity index (CCI) is a widely used tool for estimating the comorbid disease status of patients [27]. The CCI is a sum of the weighted scores of each participant’s comorbid disease based on the following 19 conditions: myocardial infarction, congestive heart failure, peripheral vascular disease, cerebrovascular disease, dementia, chronic pulmonary disease, connective tissue disease, peptic ulcer disease, mild liver disease, diabetes without end-organ damage, hemiplegia, moderate or severe renal disease, diabetes with end-organ damage, tumor without metastases, leukemia, lymphoma, moderate or severe liver disease, metastatic solid tumor, and acquired immunodeficiency syndrome (AIDS). The CCI was assessed using previously reported methods [28]. Household income was estimated by the insurance premium of each participant.

### 2.3. Statistical Analyses

The chi-squared and paired *t*-tests were used for the categorical variables and the continuous variables, respectively, to compare the general characteristics of the participants at baseline and follow-up. Linear regression analysis was performed to determine the linear relationship between body compositional changes (ΔRelative LBM, ΔRelative BFM, ΔRelative ASM, and ΔBMI) and changes in the metabolic profiles (ΔWC, ΔSBP, ΔDBP, ΔFBS, ΔTG, and ΔHDL-C). A regression analysis was performed after adjusting for variables, such as age, household income, smoking status, alcohol consumption, physical activity, and the CCI. Logistic regression analysis was then performed to determine the adjusted odds ratios (aORs) and 95% confidence intervals (CIs) of newly developed MetS and pathologies, including abdominal obesity, hyperglycemia, hypertension, hypertriglyceridemia, and low HDL-C, that were determined during follow-up based on the changes in the body composition (1% increments) between the baseline and follow-up examinations. Stratified analyses of the effects of changes in body composition on the risk of MetS were conducted according to the subgroup of the baseline BMI, baseline BFMI quartile, and the number of initial metabolic pathologies (presence of abdominal obesity, hypertension, hyperglycemia, hypertriglyceridemia, and low HDL-C).

To evaluate the combined effects of the changes in BMI and body composition, stratified analysis was performed to evaluate the effect of changes in BMI during the two health examinations on the risk of MetS according to the changes in the body composition. We categorized the participants into three groups: decreased BMI (BMI decreased by >2 kg/m^2^ during the two sequential health examinations), maintained BMI (changes in BMI between −2 kg/m^2^ and +2 kg/m^2^), and increased BMI (BMI increased by >2 kg/m^2^). We then performed a logistic regression analysis of the newly developed MetS cases according to the changes in their body composition (ΔRelative LBM, ΔRelative BFM, and ΔRelative ASM). The covariates considered in the analysis included continuous variables, age and CCI, and categorical variables, such as household income (1st, 2nd, 3rd, and 4th quartiles), physical activity (none, moderate, and vigorous), smoking status (never, ex-smoker, and current smokers), and alcohol consumption (no, moderate drinker, and heavy drinker). Statistical significance was defined as *p* < 0.05 in a two-sided manner. All data collection and analyses were conducted using SAS version 9.4 (SAS Institute Inc., Cary, NC, USA) and STATA version 13.0 (StataCorp LP, College Station, TX, USA).

## 3. Results

### 3.1. General Characteristics of the Participants

Table 1 lists the descriptive characteristics of the participants during the baseline and follow-up periods. Among the 190,599 study participants, 101,536 (53.27%) were men and 89,063 (46.73%) were women. During the two-year follow-up period, the mean BMI, WC, SBP, DBP, and the metabolic profiles, including FSG and TG, increased whereas HDL-C decreased. Furthermore, the body composition changed; the relative LBM, BFM, and ASM changed to 72.06 ± 5.97% (follow-up) from 72.13 ± 5.94% (baseline; *p* < 0.001), 26.37 ± 6.57% from 26.29 ± 6.55% (*p* < 0.001), and 30.29 ± 3.81% from 30.25 ± 3.73% (*p* < 0.001), respectively.

### 3.2. Linear Association between Body Composition Change and Changes in the Metabolic Profile

The association between the changes in the variable body composition and the changes in the metabolic profiles, WC, BP, FSG, serum TG, and serum HDL-C are shown in Table 2. In men, as relative muscle mass (LBM or ASM) increased, the metabolic profiles improved. In contrast, as the relative BFM increased, the metabolic profiles worsened. Furthermore, the change in BMI showed similar results as the change in the relative BFM. In women, all results were similar, except for the association between the relative ASM change and FSG. An increase in relative ASM did not show a significant association with FSG levels.

### 3.3. Risk of MetS and Changes in Body Composition

The multivariable logistic regression analysis revealed an association between the risk of MetS and changes in body composition (Table 3). In men, with a 1% increase in the relative LBM, the OR for developing MetS was 0.78 (95% CI, 0.77–0.79), and the ORs for a 1% increase in the relative BFM and relative ASM were 1.25 (95% CI, 1.24–1.27) and 0.61 (95% CI, 0.60–0.62), respectively. In women, the OR for relative LBM was 0.80 (95% CI, 0.79–0.81), for relative BFM was 1.24 (95% CI, 1.22–1.26), and for relative ASM was 0.61 (95% CI, 0.59–0.63). For the stratified analysis, these results and trends were similarly observed regardless of the baseline BMI status as follows: men and women who were normal weight (BMI 18.5–22.9 kg/m^2^), overweight (BMI 23–24.9 kg/m^2^), and obese (BMI ≥ 25 kg/m^2^), baseline BFMI quartile (Q1, Q2, Q3, and Q4), and the number of metabolic pathologies recorded during the baseline health examination (0, 1, and 2). The effect of increased relative LBM and ASM on the risk of MetS was more prominent when the participants were more obese (*p* for trend < 0.001), and the effect increased when the initial BFMI was higher (*p* for trend < 0.001). The fewer the initial metabolic pathologies, the greater was the effect on the reduction in the risk of MetS (*p* for trend < 0.001). Contrary to the effect of relative LBM and ASM, an increased relative BFM increased the risk of MetS in all subgroups. In women, the results showed a similar association between changes in the relative body composition and the risk of MetS.

### 3.4. Risk of Metabolic Pathologies and Body Composition Change

Table 4 shows the risks of the newly developed metabolic pathologies according to the changes in body composition. The metabolic pathologies included abdominal obesity, hyperglycemia, high BP, low HDL-C, and high TG. During the two health examinations, an increase in the relative LBM and ASM was associated with a significant risk reduction in the development of metabolic pathologies. In particular, an increase in the relative ASM showed the most prominent protective effect on the development of all metabolic pathologies. In contrast, an increased relative BFM increased the risk of metabolic pathologies. In women, all changes in the body composition profile showed the same effect on the metabolic pathologies.

### 3.5. Risk of MetS According to The Changes in BMI

Table 5 shows the effect of BMI change during the two health examinations on the risk of MetS according to the changes in the body composition. In the majority of participants, the BMI was maintained (95,750 men and 82,303 women). In men, the protective effects of the increase in body muscle mass were observed in all subgroups of BMI-change status but were most prominent in the decreased-BMI group. From these subgroup analyses, we found that even with weight gain, the risk of MetS decreased when the relative muscle mass increased. The increase in the relative BFM showed the most harmful effect on the decreased-BMI group (aOR, 1.44; 95% CI, 1.34–1.55) among all subgroups (maintained-BMI group: aOR, 1.25; 95% CI, 1.24–1.26; and increased-BMI group: aOR, 1.07; 95% CI, 1.03–1.12). Similar results were observed in women. However, no significant change in the risk was observed in the increased-BMI group in women.

## 4. Discussion

The findings of this large-scale population study suggested that an increase in relative muscle mass and a decrease in fat mass decreased the risk of MetS and improved the metabolic profiles. In particular, the same result was found in men even when BMI increased. This result suggests that, in agreement with our hypothesis, an increase in BMI may not necessarily be critical to an increase in the risk of MetS and that a change in body composition may be a more meaningful indicator for assessing the risk of MetS. In particular, the results were consistent with our hypothesis that a change in body composition, that is, an increase in relative muscle mass (LBM and ASM) or a decrease in relative BFM, lowered the risk of MetS regardless of the change in body weight.

Recently, skeletal muscle has been considered an endocrine and paracrine organ. It is well established that skeletal muscle regulates glucose metabolism through interorgan crosstalk with the stomach, liver, kidney, and brain [29]. Moreover, it is well-known that the loss of muscle mass is associated with MetS through complex factors, including insulin resistance [30], chronic inflammation [31], mitochondrial dysfunction [32], and lack of physical activity [33]. Furthermore, adiposity is a crucial factor for metabolic diseases and is strongly associated with insulin resistance [34]. Insulin resistance is the most accepted theory that explains the pathogenesis underlying the development of MetS [35]. Various pathophysiological mechanisms have been proposed to explain the connection between insulin: resistance and metabolic risk factors [36]. Our study’s findings are consistent with these concepts an increased fat mass increased the risk of MetS, and an increased muscle mass decreased the risk of MetS.

Even though several novel techniques and methods are now available for estimating body composition, BMI is still used as a surrogate marker and is assumed to be representative of adiposity. This situation is presumed to arise due to various difficulties in clinically applying new measurement methods to many subjects. A previous study showed that increased BMI is a major risk factor for MetS [37]. In general, weight gain tends to suggest increased general adiposity, but this tendency is difficult to generalize. According to the analysis of subjects with normal BMI with increased visceral fat mass, despite their normal BMI, those subjects were more insulin resistant and had more metabolic pathologies [38,39]. Moreover, obesity defined by BMI is known to be related to metabolic abnormalities, but 10% to 25% of obese subjects seemed to have a favorable metabolic profile [40]. Therefore, obesity defined by BMI alone, or weight gain in itself, may not be a proper indicator for the real adiposity related to metabolic risks. Our study’s results suggest the importance of the combined effects of the changes in BMI and body composition and highlight that the important factor in the risk of MetS is the change in body composition and not merely a change in the BMI.

In men, the increase in the relative muscle mass reduced the risk of MetS among all BMI-change (increased/decreased BMI) groups. Even if the BMI increased, the increase in relative muscle mass reduced the risk of MetS. The increase in relative BFM increased the risk of MetS among all BMI-change groups. In particular, when the BMI was reduced, the harmful effect of increased relative BFM was most prominent among BMI-change groups (*p* for trend < 0.001). In women, the maintained-BMI and the decreased-BMI groups showed similar results as in men. However, there was a difference in the increased-BMI group; the risk of MetS did not significantly decrease despite an increase in the relative muscle mass. In addition, the risk of MetS did not increase significantly with an increase in the relative BFM. A possible explanation for this result is that an increase in BMI and body weight is not achieved solely by an increase in BFM or muscle mass. If the relative muscle mass increased as the BMI decreased, it could be inferred that the BFM decreased relatively and absolutely. In addition, if the relative muscle mass increased while the BMI was maintained, we could infer that the relative fat mass decreased and the increase in the absolute fat mass was not noticeable.

In contrast, when the BMI increases, it is difficult to expect only lean muscle mass or BFM to increase in isolation. Furthermore, in women, it is difficult to expect an increase in muscle mass alone [41], particularly in postmenopausal women [42,43]. Therefore, in women with an increased BMI, there is no significant change in the risk of MetS due to an increase in relative muscle mass; therefore, it can be speculated that there might be some degree of a tradeoff effect between an increased relative muscle mass and an increased absolute BFM. In addition, among women, the amount of baseline muscle mass is relatively smaller than that in men and, therefore, even if the ratio of the relative muscle mass increases, the physiological effects on the metabolic profiles according to the increase in muscle mass described above may not be noticeable. In women with an increased BMI, an insignificant harmful effect of increasing the risk of MetS due to an increased relative BFM was also observed. It is known that body fat deposition in women tends to occur in the form of subcutaneous fat, unlike in men [44]. Subcutaneous fat deposition was found to have an inverse relationship with the risk of MetS [45,46]. Thus, it can be estimated that the effects of such subcutaneous fat deposition, increase in the overall BFM, and decrease in the relative muscle mass are mutually attenuated. However, this cannot be evaluated due to the limitations of this study dataset and must be examined through future research.

Our study has several strengths. First, to the best of our knowledge, this is the first large, population-based study to investigate the association between body composition changes and the risk of MetS. Some studies have previously addressed the association between body composition and MetS, although they have mainly focused on the relationship between body composition and MetS at a cross-sectional time point [47] or the state of body composition at one time point and the risk of developing MetS thereafter [48]. Compared with previous studies, this study is unique because it reveals how changes in body composition over a period affect the risk of MetS. Second, our study evaluated the combined effect of the changes in BMI and body composition on the risk of developing MetS. By comparing the effects of the changes in BMI, which is known as the main risk factor for MetS, and the changes in body composition, we showed that the factors affecting the risk of MetS are the changes in the body composition rather than the changes in the BMI. Especially, in men, even if the participant gained weight, his risk of MetS decreased if the weight gain was due to a gain in muscle mass. Third, ours is the first study to use the results of body composition calculated by prediction equations to evaluate the risk of MetS and the risk of metabolic pathologies. In addition, the results of these studies are consistent with previous studies that have evaluated the association between body composition and the risk of MetS [9,10,13]. When body composition is evaluated through methods such as dual-energy X-ray absorptiometry or bioelectrical impedance analysis, there may be an inevitable limitation in applying the modalities to a large population. This study is valuable as a fundamental study that can support the evaluation of metabolic risk by calculating body composition through a prediction equation in a large population.

The results of this study have practical implications. We found that evaluating body composition may be more appropriate to assess the risk of MetS than BMI alone, which is generally used as an indicator of obesity. As such, changes in body composition can be evaluated through various anthropometric measures, simple blood tests, and surveys without using complex instruments; these methods can be used in various clinical practices and epidemiological studies in the future. Furthermore, the results serve as a basis for emphasizing the equal clinical importance of increasing the muscle mass when recommending weight loss to overweight patients.

Our study has some limitations. First, the body composition was not measured by direct methods, such as dual-energy X-ray absorptiometry or bioelectrical impedance analysis. Consequently, there is a limitation that the subject’s body composition is a predicted value derived using a prediction equation rather than an actual measured value. Moreover, because of this limitation, it cannot be determined whether the change in the BFM was dominated by increased visceral fat or increased subcutaneous fat. Second, the prediction equation used in this study was not validated for patients with chronic kidney disease stage 4 and 5 with an estimated glomerular filtration rate of <30 mL/min/1.73 m^2^. Thus, our results are limited to the disease target group and cannot be extrapolated to patients with chronic kidney disease (stage 4 or 5). Third, the ethnicity of the study subjects was limited to Asians, which might limit the application of the results to all ethnic groups. Fourth, the follow-up period for observing changes in the body composition was relatively short (2 years), which may be insufficient to observe changes in the body composition and the risk of MetS. This study confirmed some degrees of changes in the risk of MetS according to the changes in the body composition, although a more remarkable result could have been confirmed if the follow-up period was longer.

## 5. Conclusions

In conclusion, in Korean adults, a 1% increase in the relative LBM decreased the risk of MetS by 19–21%, and a 1% increase in the relative ASM reduced the risk of MetS by approximately 38%. Further, a 1% increase in the relative BFM increased the risk of MetS by 24–25%. This result was more prominent when the baseline BMI or BFMI was higher. In addition, in men, even if the BMI increased, the risk of MetS decreased as the relative muscle mass increased. The benefits and risks of increased relative muscle mass and BFM were attenuated when the BMI was increased in women. Based on our results, we recommend focusing on increasing the relative muscle mass and decreasing BFM rather than just decreasing BMI, especially in obese individuals. Further study is needed to evaluate the tradeoff between relative muscle mass gain and absolute body fat gain.

## Figures and Tables

**Table 1:** Descriptive characteristics of the study participants (*n* = 190,599).

Variable	Initial	Follow-Up	*p*-Value
Age, years, mean (SD)	58.73 ± 8.26	60.73 ± 8.26	<0.001
Sex, *n* (%)			1.000
Men	101,536 (53.27)	101,536 (53.27)	
Women	89,063 (46.73)	89,063 (46.73)	
Smoking, *n (%)*			<0.001
Never	124,513 (65.33)	124,552 (65.35)	
Ex-smoker	36,328 (19.06)	38,746 (20.33)	
Current smoker	29,758 (15.61)	27,301 (14.32)	
Alcohol use, *n (%)*			<0.001
No	28,315 (14.86)	27,553 (14.46)	
Moderate drinker	31,605 (16.58)	32,188 (16.89)	
Heavy drinker	130,679 (68.56)	130,858 (68.66)	
Physical activity, *n (%)*			<0.001
None	101,270 (53.13)	98,484 (51.67)	
Moderate	71,468 (37.50)	73,348 (38.48)	
Vigorous	17,861 (9.37)	18,767 (9.85)	
BMI, kg/m^2^, mean (SD)	23.53 ± 2.42	23.55 ± 2.47	<0.001
WC, cm, mean (SD)	80.03 ± 7.06	80.46 ± 7.40	<0.001
Serum creatinine, mg/dL (SD)	0.89 ± 0.19	0.87 ± 0.19	<0.001
FSG, mg/dL (SD)	95.96 ± 18.89	97.89 ± 19.58	<0.001
TG, mg/dL (SD)	113.74 ± 63.34	119.92 ± 71.39	<0.001
HDL-C, mg/dL (SD)	56.78 ± 16.22	56.01 ± 17.63	<0.001
SBP, mm Hg (SD)	122.71 ± 14.46	123.30 ± 14.40	<0.001
DBP, mm Hg (SD)	76.27 ± 9.56	76.39 ± 9.53	<0.001
Taking antihypertensive, *n* (%)	68,458 (35.92)	76,260 (40.01)	<0.001
Taking OHA, *n* (%)	10,545 (5.53)	12,599 (6.61)	<0.001
Predicted LBM index (kg/m^2^)	16.92 ± 1.77	16.91 ± 1.78	<0.001
Predicted ASM index (kg/m^2^)	7.10 ± 1.01	7.11 ± 1.03	<0.001
Predicted BFM index (kg/m^2^)	6.24 ± 1.88	6.27 ± 1.91	<0.001
Relative LBM (%)	72.13 ± 5.94	72.06 ± 5.97	<0.001
Relative BFM (%)	26.29 ± 6.55	26.37 ± 6.57	<0.001
Relative ASM (%)	30.25 ± 3.73	30.29 ± 3.81	<0.001

Values are represented as the mean (M) ± standard deviation (SD) or number (%). *p*-values calculated using the chi-squared test for categorical variables and paired *t*-test for continuous variables. BMI, body mass index; WC, waist circumference; SBP, systolic blood pressure; DBP, diastolic blood pressure; FSG, fasting serum glucose; TG, triglyceride; HDL-C, high-density lipoprotein cholesterol, OHA, oral hypoglycemic agent; LBM, lean body mass; BFM, body fat mass; ASM, appendicular skeletal muscle mass.

**Table 2:** Multiple linear regression models for the predictors of metabolic profiles.

Variable	ΔWC	ΔSBP	ΔDBP	ΔFSG	ΔTG	ΔHDL-C
	*β* (95% CI)	*β* (95% CI)	*β* (95% CI)	*β* (95% CI)	*β* (95% CI)	*β* (95% CI)
**Men**						
ΔRelative LBM	−2.56 (−2.57, −2.56)	−0.66 (−0.72, −0.61)	−0.40 (−0.44, −0.36)	−0.28 (−0.35, −0.21)	−4.85 (−5.11, −4.58)	0.50 (0.43, 0.57)
ΔRelative BFM	2.51 (2.50, 2.51)	0.67 (0.62, 0.72)	0.40 (0.36, 0.43)	0.21 (0.14−0.28)	4.65 (4.39, 4.92)	−0.51 (−0.57, −0.44)
ΔRelative ASM	−5.32 (−5.34, −5.31)	−1.24 (−1.34, −1.13)	−0.75 (−0.83, −0.67)	−0.47 (−0.62, −0.32)	−9.26 (−9.81, −8.70)	1.00 (0.86−1.13)
ΔBMI	1.61 (1.58, 1.64)	1.31 (1.22, 1.39)	0.76 (0.71, 0.82)	0.31 (0.20−0.42)	9.54 (9.12, 9.96)	−1.01 (−1.11, −0.91)
**Women**						
ΔRelative LBM	−2.64 (−2.66, −2.62)	−0.82 (−0.89, −0.75)	−0.40 (−0.45, −0.35)	−0.20 (−0.27, −0.12)	−4.38 (−4.67, −4.09)	0.44 (0.34, 0.53)
ΔRelative BFM	2.67 (2.65, 2.69)	0.85 (0.77, 0.92)	0.41 (0.36, 0.46)	0.21 (0.14−0.29)	4.58 (4.28, 4.88)	−0.45 (−0.55, −0.35)
ΔRelative ASM	−6.03 (−6.09, −5.98)	−2.06 (−2.24, −1.88)	−0.93 (−1.05, −0.80)	−0.10 (−0.29, 0.08)	−10.28 (−11.01, −9.55)	1.29 (1.05−1.5)
ΔBMI	1.51 (1.48, 1.54)	0.96 (0.88, 1.05)	0.49 (0.43, 0.55)	0.32 (0.24−0.41)	5.21 (4.88, 5.54)	−0.46 (−0.57, −0.35)

Linear regression analysis after adjustments for age, household income, smoking, alcohol consumption, physical activity, and the Charlson comorbidity index. WC, waist circumference; SBP, systolic blood pressure; DBP, diastolic blood pressure; FSG, fasting serum glucose; TG, triglyceride; HDL-C, high-density lipoprotein cholesterol, LBM, lean body mass; BMI, body mass index; ASM, appendicular skeletal muscle mass; BFM, body fat mass.

**Table 3:** Adjusted odds ratios and 95% confidence intervals of metabolic syndrome according to the changes (1% increments) in multiple body composition parameters in the subgroups.

Men	Subjects, *n*	Events, *n* (%)	ΔRelative LBM	ΔRelative BFM	ΔRelative ASM
All male participants	101,536	18,006 (17.73)	0.78 (0.77, 0.79)	1.25 (1.24, 1.27)	0.61 (0.60, 0.62)
BMI category					
Normal	41,344	4029 (9.75)	0.82 (0.80, 0.83)	1.20 (1.18, 1.22)	0.69 (0.66, 0.71)
Overweight	33,179	5717 (17.23)	0.72 (0.71, 0.73)	1.36 (1.34, 1.39)	0.54 (0.52, 0.56)
Obese	27,013	8260 (30.58)	0.66 (0.65, 0.68)	1.47 (1.44, 1.50)	0.46 (0.44, 0.48)
*p* for trend			<0.001	<0.001	<0.001
BFMI quartile					
Q1	25,384	1990 (7.84)	0.79 (0.77, 0.81)	1.24 (1.21, 1.27)	0.65 (0.61, 0.68)
Q2	25,384	3347 (13.19)	0.67 (0.66, 0.69)	1.45 (1.42, 1.49)	0.47 (0.44, 0.49)
Q3	25,384	4898 (19.30)	0.58 (0.56, 0.59)	1.71 (1.67, 1.75)	0.35 (0.33, 0.36)
Q4	25,384	7771 (30.61)	0.58 (0.56, 0.59)	1.69 (1.65, 1.73)	0.34 (0.33, 0.36)
*p* for trend			<0.001	<0.001	<0.001
No. of metabolic pathologies recorded in the baseline health exam					
0	18,117	869 (4.80)	0.72 (0.69, 0.75)	1.36 (1.31, 1.41)	0.51 (0.47, 0.56)
1	39,773	4803 (12.08)	0.74 (0.73, 0.75)	1.32 (1.30, 1.35)	0.55 (0.53, 0.57)
2	43,646	12,334 (28.26)	0.76 (0.75, 0.77)	1.29 (1.28, 1.31)	0.57 (0.56, 0.59)
*p* for trend			<0.001	<0.001	<0.001
**Women**					
All women participants	89,063	14,858 (16.68)	0.80 (0.79−0.81)	1.24 (1.22−1.26)	0.61 (0.59−0.63)
Initial BMI category					
Normal	41,497	4104 (9.89)	0.82 (0.80, 0.84)	1.21 (1.19, 1.24)	0.64 (0.60, 0.68)
Overweight	25,511	4380 (17.12)	0.72 (0.70, 0.74)	1.39 (1.36, 1.43)	0.48 (0.45, 0.52)
Obese	21,883	6374 (29.00)	0.67 (0.65, 0.68)	1.50 (1.46, 1.55)	0.41 (0.38, 0.44)
*p* for trend			<0.001	<0.001	<0.001
Initial BFMI quartile					
Q1	22,266	1701 (7.64)	0.83 (0.80, 0.85)	1.21 (1.17, 1.25)	0.65 (0.60, 0.70)
Q2	22,266	2688 (12.07)	0.75 (0.73, 0.77)	1.33 (1.29, 1.38)	0.52 (0.48, 0.56)
Q3	22,266	3976 (17.86)	0.68 (0.66, 0.70)	1.47 (1.43, 1.52)	0.41 (0.38, 0.44)
Q4	22,266	6493 (29.16)	0.64 (0.62, 0.66)	1.56 (1.51, 1.60)	0.36 (0.34, 0.39)
*p* for trend			<0.001	<0.001	<0.001
No. of metabolic pathologies recorded in the baseline health exam					
0	20,928	811 (3.88)	0.72 (0.68, 0.76)	1.290 (1.32, 1.47)	0.48 (0.42, 0.54)
1	35,743	4280 (11.97)	0.78 (0.76, 0.80)	1.28 (1.25, 1.31)	0.57 (0.53, 0.60)
2	32,392	9767 (30.15)	0.78 (0.76, 0.79)	1.28 (1.26, 1.30)	0.56 (0.54, 0.59)
*p* for trend			<0.001	<0.001	<0.001

The odds ratios are calculated using logistic regression analysis after adjustments for age, household income, physical activity, smoking, alcohol intake, and the Charlson comorbidity index score. BMI category: normal, <23 kg/m^2^; overweight, 23–24.9 kg/m^2^; obese, ≥25 kg/m^2^. BMI, body mass index; LBM, lean body mass; BFM, body fat mass; ASM, appendicular skeletal muscle mass; BFMI, body fat mass index; Q, quartile.

**Table 4:** Adjusted odds ratios and 95% confidence intervals of the newly developed metabolic paTable 1. increments) in multiple body composition parameters.

**Men**	**Participants, *n***	**Events, *n* (%)**	**ΔRelative LBM**	**ΔRelative BFM**	**ΔRelative ASM**
Waist ≥ 90 cm	90,895	9148 (10.06)	0.50 (0.49, 0.51)	1.95 (1.92, 1.98)	0.23 (0.22, 0.24)
Hyperglycemia	69,355	18,633 (26.87)	0.94 (0.93, 0.95)	1.04 (1.03, 1.05)	0.90 (0.88, 0.92)
High BP	44,191	16,380 (37.07)	0.92 (0.91, 0.93)	1.07 (1.06, 1.08)	0.85 (0.83, 0.87)
Low HDL-C	95,127	8717 (9.16)	0.94 (0.93, 0.95)	1.05 (1.04, 1.06)	0.90 (0.88, 0.93)
High TG	81,047	15,030 (18.54)	0.88 (0.87, 0.89)	1.12 (1.11, 1.13)	0.80 (0.78, 0.81)
**Women**	**Subjects, *n***	**Events, *n* (%)**	**ΔRelative LBM**	**ΔRelative BFM**	**ΔRelative ASM**
Waist ≥ 85 cm	79,332	8407 (10.60)	0.53 (0.52, 0.54)	1.86 (1.82, 1.89)	0.25 (0.24, 0.26)
Hyperglycemia	71,159	13,657 (19.19)	0.97 (0.95, 0.98)	1.03 (1.01, 1.04)	0.96 (0.93, 1.00)
High BP	43,154	14,093 (32.66)	0.93 (0.92, 0.95)	1.06 (1.05, 1.08)	0.86 (0.83, 0.89)
Low HDL-C	72,799	13,469 (18.50)	0.96 (0.94, 0.97)	1.04 (1.02, 1.05)	0.90 (0.87, 0.93)
High TG	78,344	11,684 (14.91)	0.88 (0.87, 0.89)	1.13 (1.11, 1.15)	0.76 (0.73, 0.79)

The odds ratios are calculated using logistic regression analysis after adjustments for age, household income, physical activity, smoking, alcohol intake, and the Charlson comorbidity index score. HDL-C, high-density lipoprotein cholesterol; TG, triglyceride; BP, blood pressure.

**Table 5:** Adjusted odds ratios and 95% confidence intervals for metabolic syndrome according to multiple predictors (1% increments) and body mass index change.

**Men**	**Participants**	**Events**	**ΔRelative LBM**	**ΔRelative BFM**	**ΔRelative ASM**
**BMI change**					
Decreased	2848	296 (10.39)	0.68 (0.63, 0.73)	1.44 (1.34, 1.55)	0.47 (0.40, 0.54)
Maintained	95,750	16,767 (17.51)	0.78 (0.78, 0.79)	1.25 (1.24, 1.26)	0.62 (0.61, 0.63)
Increased	2938	943 (32.10)	0.92 (0.88, 0.96)	1.07 (1.03, 1.12)	0.80 (0.73, 0.88)
*p* for trend			<0.001	<0.001	<0.001
**Women**	**Subjects**	**Events**	**ΔRelative LBM**	**ΔRelative BFM**	**ΔRelative ASM**
**BMI change**					
Decreased	3393	501 (14.77)	0.71 (0.65, 0.78)	1.40 (1.28, 1.54)	0.49 (0.40, 0.61)
Maintained	82,303	13,358 (16.23)	0.78 (0.77, 0.80)	1.28 (1.26, 1.30)	0.61 (0.58, 0.63)
Increased	3367	999 (29.67)	1.04 (0.97, 1.11)	0.93 (0.87, 1.00)	1.12 (0.95, 1.31)
*p* for trend			<0.001	<0.001	<0.001

Maintained BMI: body mass index changes between −2 kg/m^2^ and +2 kg/m^2^ as compared with baseline BMI. The odds ratios are calculated using logistic regression analysis after adjustments for age, household income, physical activity, smoking, alcohol intake, and the Charlson comorbidity index score. BMI, body mass index; LBM, lean body mass; BFM, body fat mass; ASM, appendicular skeletal muscle mass.

## Supplementary Materials

The following are available online at https://www.mdpi.com/2077-0383/10/4/745/s1, Table S1: Adapted prediction equations for lean body mass, body fat mass, and appendicular skeletal muscle mass among adults.
