# Supplementary material for: Changes in Body Composition Are Associated with Metabolic Changes and the Risk of Metabolic Syndrome
Source: J Clin Med. 2021 Feb 13;10(4):745. doi: 10.3390/jcm10040745 (PMC7918782; doi:10.3390/jcm10040745)
Supplement: Supplementary file 1 [file jcm-10-00745-s001.pdf]

Supplementary data. Adapted prediction equations for lean body mass, body fat mass, and appendicular skeletal muscle mass among adults

1. Men

- LBMI=7.838–0.001*Age+0.667*BMI-0.065*WC-0.140*sCr+0.093*PA_mod+0.332*PA_vig-0.123*Smk_ex+0.098*Smk_cur+ 0.036*Alc_mod+0.121*Alc_heavy

- BFMI=-7.715+0.001*Age+0.313*BMI+0.066*WC+0.137*sCr-0.083*PA_mod-0.305*PA_vig+0.174*Smk_ex-0.106*Smk_cur-0.033*Alc_mod-0.124*Alc_heavy

- ASMI=3.970-0.010*Age+0.303*BMI-0.033*WC-0.016*sCr+0.081*PA_mod+0.231*PA_vig-0.022*Smk_ex-0.01*Smk_cur+0.060*Alc_mod+0.085* Alc_heavy

2. Women

- LBMI=6.149-0.004*Age+0.456*BMI-0.023*WC+0.136*sCr+0.052*PA_mod+0.272*PA_vig-0.093*Smk_ex+0.081*Smk_cur+0.066*Alc_mod+0.090* Alc_heavy

- BFMI=-6.075-0.003*Age+0.535*BMI-0.021*WC-0.108*sCr-0.018*PA_mod-0.223*PA_vig+0.110*Smk_ex-0.095*Smk_cur-0.067*Alc_mod-0.091*Alc_heavy

- ASMI=2.434-0.004*Age+0.187*BMI-0.008*WC+0.222*sCr+0.038*PA_mod+0.165*PA_vig-0.058*Smk_ex-0.012*Smk_cur+0.040*Alc_mod+0.014*Alc_heavy

Abbreviations: LBMI, lean body mass index; BFMI, body fat mass index; ASMI, appendicular skeletal muscle mass index; BMI, body mass index; WC, waist circumference; SCr, serum creatinine; PA_mod, moderate physical activity; PA_vig, vigorous physical activity; Smk_ex, ex-smokers; Smk_cur, current smokers; Alc_mod, moderate alcohol consumption; Alc_heavy, heavy alcohol consumption
